# Supplementary material for: Rapid Absorption of Naloxone from Eye Drops
Source: Pharmaceuticals (Basel). 2022 Apr 25;15(5):532. doi: 10.3390/ph15050532 (PMC9143859; doi:10.3390/ph15050532)
Supplement: Supplementary file 1 [file pharmaceuticals-15-00532-s001.zip › pharmaceuticals-1686595-supplementary.pdf]

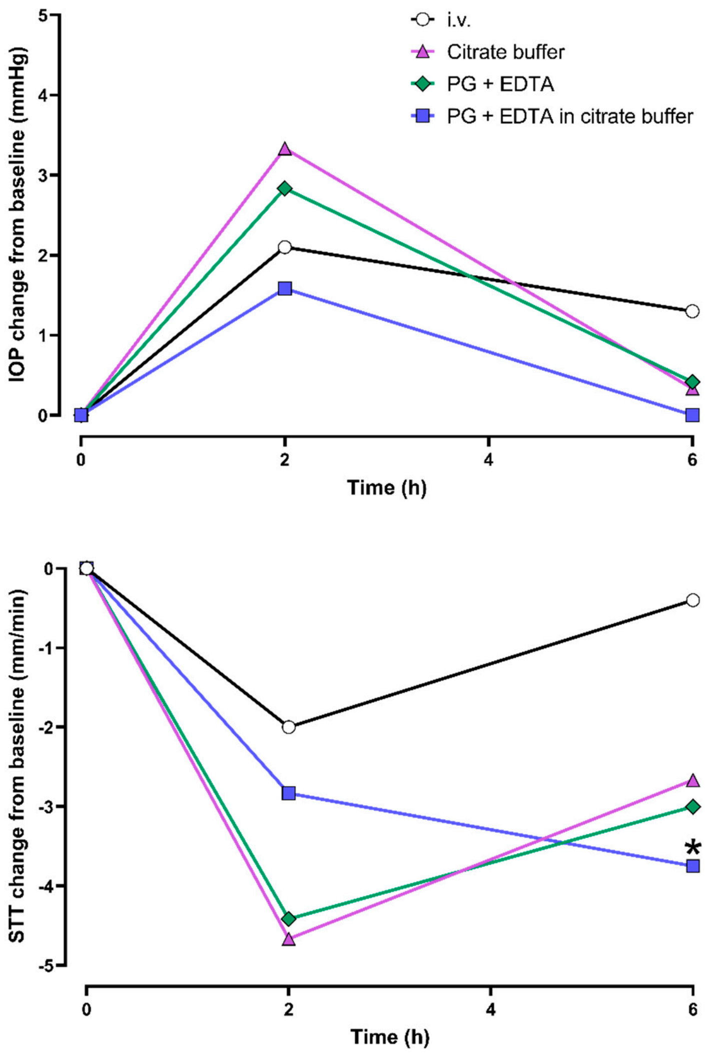

Supplemental Figure S1: Change from baseline in intraocular pressure (upper panel) and tear production (lower panel) after either intravenous or ocular administration of 100 µg/kg naloxone to 6 male dogs.

EDTA, ethylenediaminetetraacetic acid; IOP, intraocular pressure; i.v., intravenous; PG, propylene glycol; STT, Schirmer's tear test.

*P = 0.02 compared to i.v. administration.
